# Comparison of Upper Airway Ultrasonography With End-Tidal Capnography for the Confirmation of Endotracheal Tube Placement in Patients Requiring General Anesthesia

**DOI:** 10.7759/cureus.35642

**Published:** 2023-03-01

**Authors:** Keerthana Shivaji, Sridevi Mulimani, Deepa S D, Anusha Suntan

**Affiliations:** 1 Anesthesiology, Shri BM Patil Medical College and Hospital, BLDE University, Vijayapura, IND

**Keywords:** general anesthesia, intubation, capnography, upper airway ultrasonography, endotracheal tube

## Abstract

Introduction

End-tidal capnography (EtCO_2_) has been the gold standard method for confirmation of endotracheal intubation. Upper airway ultrasonography (USG) is a new promising method for confirming endotracheal tube (ETT) placement and has the potential to become the first-line non-invasive airway assessment tool in the future thanks to widespread POCUS knowledge, greater technology improvements, portability, and availability of ultrasound in the majority of essential areas. Hence our study aimed to compare upper airway USG and EtCO_2_ for the confirmation of ETT placement in patients undergoing general anesthesia.

Aim

To compare the upper airway USG with EtCO_2_ for confirmation of ETT placement in patients requiring general anesthesia for elective surgical procedures. The objectives of the study were to compare the time taken for confirmation, and the number of correct identification of tracheal and esophageal intubation by both upper airway USG and EtCO_2_.

Materials and methods

After obtaining institutional ethical committee (IEC) approval, a prospective randomized comparative study involving 150 patients under American Society of Anesthesiologists (ASA) grade I and II requiring endotracheal intubation for elective surgeries under general anesthesia was randomized into two groups, Group U-upper airway USG and Group E-EtCO_2 _with 75 patients in each group. ETT placement confirmation was done by upper airway USG in Group U and by EtCO_2 _in Group E and the time taken for confirmation of ETT placement and correct identification of esophageal and tracheal intubation by USG and EtCO_2 _was noted.

Results

The demographic details among both groups were statistically insignificant. Upper airway USG had a faster average confirmation time of 16.41 seconds when compared to EtCO_2_ which took an average confirmation time of 23.56 seconds. In our study, upper airway USG was able to identify esophageal intubation with 100% specificity.

Conclusion

Upper airway USG can be a reliable method and can be employed as a standard method for confirmation of ETT location in patients undergoing elective surgeries under general anesthesia when compared to EtCO_2_.

## Introduction

Endotracheal intubation for airway protection with an endotracheal tube (ETT) is a key competency in anesthesia, emergency, and critical care. It can be disastrous if an ETT placement cannot be properly confirmed in a pharmacologically paralyzed patient (as in an operating room). Esophageal intubation has been associated with an increase in morbidity and mortality. Direct visualization of the ETT passing through the vocal cords is not always feasible, especially in the case of difficult laryngoscopy [1]. The Gold standard method to quickly determine the location of an ETT has been the use of capnography (EtCO_2_) [2]. In some low pulmonary flow conditions, such as cardiac arrest or severe shock, the validity of quantitative capnography with respect to confirmation of ETT placement is criticized where Upper airway ultrasonography (USG) images will not be affected under these conditions. In order to confirm the insertion of the ETT in such circumstances, upper airway USG may be utilized [3].

USG is an emerging tool for airway management and has the potential for application in peri-operative airway management in the future. There are numerous studies that support the upper airway USG is helpful in confirming the placement of ETT in both elective and emergency settings. The application of upper airway USG for ETT placement in elective clinical procedures will aid in improved accuracy because of real-time imaging of the passage of ETT through the vocal cords and therefore reduce the peri-operative complications associated with esophageal intubation. The advantages of USG are that it is light, portable, non-invasive, has reproducible images, is widely available, painless, reasonably priced, easily repeatable, and has a solid safety profile in use by non-radiologists with a relatively quick learning curve [4,5].

This study's aim was to determine whether USG can be a reliable and quicker method than EtCO_2_ for verifying ETT placement in individuals undergoing elective procedures under general anesthesia. The primary and secondary objective of this study is to compare Upper airway USG with EtCO_2_ in terms of time taken for confirmation of ETT placement and the correct identification of endotracheal and esophageal intubation.

## Materials and methods

Study design

This was a prospective, randomized, comparative study conducted from December 2020 to August 2022 in the Department of Anesthesiology, BLDE Deemed to be University (DU), Shri BM Patil Medical College, Hospital and Research Center, Vijayapura, Karnataka, India after obtaining the approval of Institutional Ethical Committee - BLDE (DU) (approval letter: IEC/No-09/2021). Inclusion criteria were American Society of Anesthesiologists (ASA) grades I-II, between the age group of 20 and 60 years, and patients requiring endotracheal intubation for elective surgeries under general anesthesia. Exclusion criteria were patients posted for neck surgeries under general anesthesia, obese patients with body mass index (BMI) more than or equal to 30.0 kilogram/metre^2 ^(kg/m^2^), patients with abnormal neck anatomy, poor pulmonary compliance, and pregnant females. Informed consent was obtained from all the patients included in the study.

Sample size

The anticipated Mean±SD (standard deviation) of time taken for confirmation of ETT placement by USG was 16.4±7.3 seconds respectively according to the study by Adi et al. [4], the required minimum sample size is 75 per group (i.e., a total sample size of 150 assuming equal group sizes) to achieve a power of 80% and a level of significance of 5% (two-sided), for detecting a true difference in means between two groups. The formula used was N= $2 \left [\frac{Z_{\alpha } +Z_{\beta }*S }{d} \right ]^{2}$, where Z_$\alpha$_=95% which is level of significance, Z_$\beta$_=80%, which is the power of the study, d=clinically significant difference between two parameters, SD=common standard deviation.

Procedure

Patients were kept nil by mouth (NBM) before surgery according to the American Society of Anesthesiologists (ASA) guidelines. ASA guidelines are two hours for clear liquids, four hours human milk, six hours for light meal, and eight hours for fatty food. When the patients were shifted to the pre-operative room, randomization was done by making envelopes for either group U (upper airway USG) and group E (EtCO_2_) and asking the patient to pick one. Patients were taken to the operation theatre, and standard monitoring devices including a pulse oximeter, non-invasive blood pressure (NIBP), and electrocardiogram (ECG) leads were connected and baseline values were recorded. The intravenous (IV) line was secured with an 18G/20G IV cannula and IV fluid was started. Patients were premedicated with injection (inj) ondansetron (0.1mg/kg) IV, inj glycopyrrolate (0.01 mg/kg) IV, inj midazolam (0.08 mg/kg) IV and inj fentanyl (2mcg/kg) IV. Pre-oxygenation was done with 100% oxygen for three minutes. Induction was done with inj propofol (2mg/kg) IV. To facilitate the ETT insertion, muscle relaxation was achieved by inj atracurium (0.6 mg/kg) IV and using direct laryngoscopy with Macintosh blade 3 for females and Macintosh blade 4 for males, the patient's trachea was intubated.

In group U, the ETT placement confirmation was done using upper airway USG (SONOSITE M - Turbo machine in B mode) by placing a linear transducer 8-12 Megahertz (MHz) on the anterior part of the neck over the suprasternal notch. Upper airway USG was done when the patients were being intubated with their head and neck in sniffing posture. In case of tracheal intubation, two hyperechoic parallel lines were seen [6]. With no change in tracheal imaging, if there is a distended esophagus with lumen appearing as a hypo-echoic shadow, it was identified as esophageal intubation. This characteristic appearance is called a double trachea sign [7].

In group E, confirmation of ETT placement was done by observing EtCO_2_ levels of 35-45 mm Hg after 5 or more breaths and detection of characteristic CO_2_ waveform [6]. The time taken for confirmation of ETT placement and the correct identification of esophageal and tracheal intubation by upper airway USG and EtCO_2_ were noted.

Soon after the confirmation, the ETT cuff was inflated with air. The patient's chest was auscultated for bilateral equal air entry and ETT was fixed appropriately. The ETT was connected to the ventilator circuit and age, weight adjusted ventilator parameters were set. Intraoperatively the patient was monitored with standard monitoring devices. Anesthesia was maintained with oxygen (O_2_) 50%, nitrous oxide (N_2_0) 50% and isoflurane 0.8%. Muscle relaxation was maintained with inj atracurium (0.08 mg/kg) IV bolus doses every 20 minutes. Patients were extubated after adequate reversal of muscle relaxation with inj glycopyrrolate (0.01 mg/kg) IV and inj neostigmine (0.05mg/kg) IV. Post extubation, the patient was shifted to Post Anesthesia Recovery Unit (PACU) and monitored for post operative complications such as fall in saturation, hypertension/hypotension, etc., with pulse oximeter, ECG, and NIBP and managed accordingly.

Statistical analysis

The information obtained was entered into a Microsoft Excel sheet, and statistical analysis was performed using a statistical package for the social sciences (Version 20). Independent t-tests were used to compare normally distributed continuous variables between two groups. Mann-Whitney U test was employed for variables that were not normally distributed. Using the Chi-square categorical variables between the two groups were compared. P-value < 0.05 was considered statistically significant. All statistical tests were performed two tailed. Results were presented as mean, (Median) ±SD, counts and percentages and diagrams.

## Results

**Figure 1:**
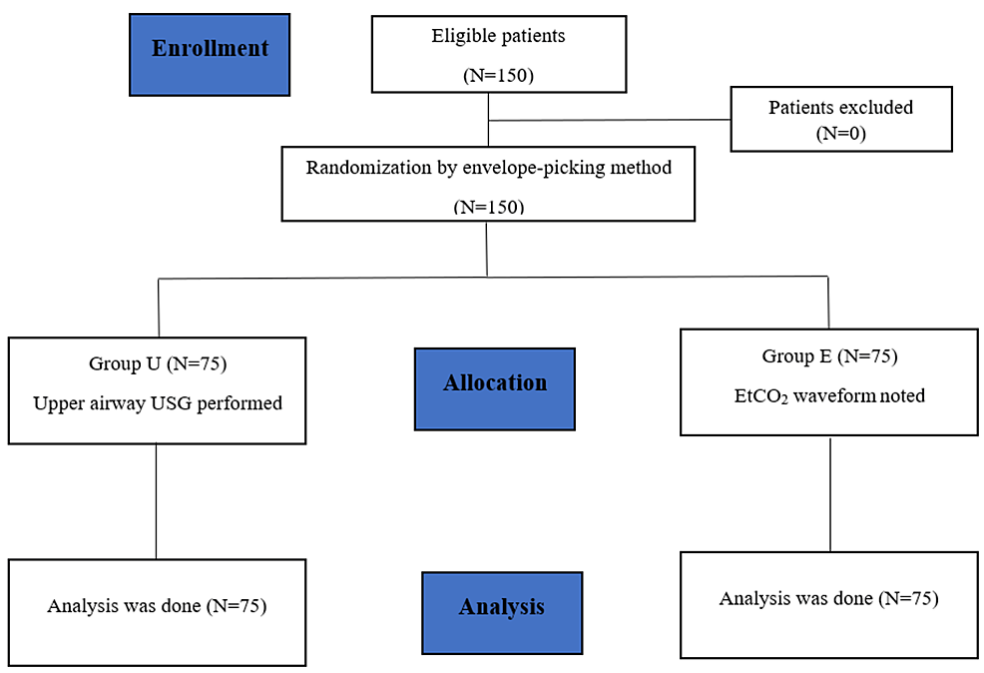
CONSORT flow diagram CONSORT=Consolidated standards of reporting trials, N=number of patients, group U=upper airway ultrasonography, group E=EtCO_2_

This prospective study included a sample size of 150 patients randomized into two groups - group U and group E by envelope picking method. Figure 1 shows the Consolidated Standards of Reporting Trials (CONSORT) diagram depicting how patients were enrolled in our study.

Table 1 shows the comparison of age between group U and group E. The different age groups included in the study such as <20, 20-29, 30-39, 40-49, 50-59, and 60 years had a percentage of 4%, 33.3%, 25.3%, 16%, 14.7%, and 6.7% in group U and 2.7%, 14.7%, 28%, 33.3%, 21.3%, and 0% in group. There was no statistical difference between group U and group E with respect to age groups. Table 2 shows the comparison of gender between group U and group E. The percentage of males and females in group U were 44% and 56% and in group E were 34.7% and 65.3%. No statistical significance was found between both the groups in terms of gender. The comparison of BMI among group U and group E was not statistically significant. The mean BMI of group U was 26.92 and mean BMI of group E was 25.79 as shown in Table 3.

**Table 1:** Comparison of age between group U and group E N=number of patients, %=percentage of patients

Age (Years)	Group U		Group E	
	N	%	N	%
< 20	3	4	2	2.7
20 - 29	25	33.3	11	14.7
30 - 39	19	25.3	21	28
40 - 49	12	16	25	33.3
50 - 59	11	14.7	16	21.3
60	5	6.7	0	0
Total	75	100	75	100
Mean±SD	36.16	12.73	40.08	10.954
Independent t-test	t=2.0, P value>0.06			

**Table 2:** Comparison of gender between group U and group E N=number of patients, %=percentage of patients

Gender	Group U		Group E	
	N	%	N	%
Female	33	44.0	26	34.7
Male	42	56.0	49	65.3
Total	75	100.0	75	100.0
Chi-square test	Chi-square test=1.369, P value=0.2420			

**Table 3:** Comparison of BMI between group U and group E BMI=Body Mass Index, SD=Standard deviation

Variable	Group U		Group E	
	Mean	SD	Mean	SD
BMI	26.92	12.390	25.79	4.262
Mann-Whitney U test	Mann-Whitney U test=2741.000, P value=0.787			

The clinical diagnosis and procedure among both groups, in which group U had the percentage of ENT, General surgery, Neurosurgery, and Urology were 66.7%, 10.7%, 6.7%, and 16%, respectively, and in group E, the percentage of ENT, General surgery, Neurosurgery, and Urology were 14.7%, 18.7%, 48%, and 18.7%, respectively, and it was statistically insignificant.

The time taken for the upper airway USG and EtCO_2 _for the confirmation of ETT placement were 16.4133 seconds and 23.56 seconds (Table 4). This is statistically significant as USG had shown to be taking less time for the identification of ETT than EtCO_2_. In our study, the Upper airway USG took 15 seconds to diagnose one esophageal intubation in real time which implies 100% specificity of upper airway USG for identification of esophageal intubation.

**Table 4:** Comparison of time taken for the verification of ETT placement by group U and group E N=number of patients Statistically significant as p-value is less than 0.05.

Variables	N	Average time taken for verification (seconds)	Mann-Whitney U test	P-value
Group U	75	16.4133	0.000	0.000
Group E	75	23.5600		

## Discussion

There are various methods to verify tracheal and esophageal intubation in elective and emergency settings. Before securing the ETT, preliminary confirmation procedures include EtCO_2_ measurement, auscultation of breath sounds, bilateral chest rise, presence of condensed vapor in the ETT, and direct observation of the ETT passing through the glottis [8,9]. This prospective study was carried out to determine whether the upper airway USG can be a quicker and a reliable method in comparison to EtCO_2_ in the verification of ETT placement.

According to the study done by Galcinao et al. [10] for determining the effectiveness of USG in confirming the ETT placement in pediatric patients, they demonstrated that sniffing posture allowed for the best picture collection. Similar to other studies, our study found that the sniffing posture was ideal for both intubation and USG of the upper airway. 

In a study done by Kristensen et al. [11], imaging superficial airway structures were best done with a linear high-frequency transducer by positioning the ultrasonic probe transversely on the neck at the level of the suprasternal notch during intubation. Davis et al. [12] undertook a cadaveric study where a low-frequency curvilinear probe was placed over the cricothyroid region in the longitudinal plane to confirm the placement of the ETT. They came to the conclusion that the dynamic trans-cricothyroid ultrasound is a reliable method of ETT confirmation following intubation after comparing the dynamic and static images of the ultrasound. Whereas in Bansal et al. [6] study, the probe was positioned horizontally from the cricothyroid membrane up to the suprasternal notch. In our study, a linear high-frequency probe was positioned transversely at the level of the suprasternal notch to observe the endotracheal intubation. USG can be utilized to directly observe whether the ETT enters the trachea or the esophagus and verify intubation without requiring ventilation. Our analysis showed the same sensitivity and specificity as the tracheal rapid ultrasound examination (TRUE) study by Masoumi et al. [13], in which they placed a convex transducer probe transversely above the suprasternal notch.

The operational characteristics value for the prediction of tracheal ETT insertion was found to be satisfied with both dynamic and static ultrasonography in the study by Saeed Abbasi et al. [14] on direct ultrasound methods. We did USG during and after intubation in our trial as well and found that both methods were satisfactory. Chou et al. [15] conducted a study in 112 patients who underwent intubation in emergency conditions such as cardiorespiratory arrest, respiratory failure, and severe trauma and was intended to evaluate the diagnostic accuracy and turnaround time for tracheal rapid ultrasound examination (TRUE) confirmation. When intubating a patient in an emergency, a low-frequency ultrasonography probe was positioned slightly above the suprasternal notch to confirm the location of the ETT. TRUE showed a sensitivity of 98.9%, a specificity of 94.1%, and an average time of 9 seconds (s). The aforementioned approaches had greater concordance, and it was discovered that TRUE could be used to quickly determine the location of an ETT in emergency intubation. Our study had shown that the upper airway USG confirmed the ETT placement with a mean average time of 16.4133 s.

In a study by Sustic et al. [4], it was discovered that two of the false negative patients had subcutaneous emphysema as a result of pneumothorax, which made it challenging to identify two hyperechoic lines. The false negative subjects were discovered to be overweight or obese with more subcutaneous fat in the neck region in a study by Kundra et al. [16]. Since patients with BMI more than equal to 30.0 kg/m^2 ^were excluded from our study, the correlation between obesity and false negative outcomes was not observed.

Pfeiffer et al. [1] conducted a study to compare the duration of bilateral lung USG with auscultation and EtCO_2_ for verifying endotracheal intubation. They came to the conclusion that USG is quicker than EtCO_2_ and the standard method of auscultation. Upper airway USG and EtCO_2_ have a rapid mean time of 12 s and 9 s, respectively, according to a study by Abhishek et al. [3]. In the study by Thomas et al. [17], ETT placement was identified more quickly by USG than by EtCO_2_ and other standard clinical techniques. In a study by Sethi et al. [18], the time taken by three USG techniques for confirmation of ETT placement was compared with that for capnography. They found that upper airway USG which took a mean time of 3.8 s, 12.1 s, and 13.81 s was faster than EtCO_2 _which took a mean time of 22.3 s, 22.3 s, and 22.1 s. Our study found that upper airway USG identified the placement of the ETT faster than EtCO_2_, with quick mean times of 16.4133 s and 23.56 s, respectively. Adi et al. [19] demonstrated a strong degree of agreement between the two modalities in their study comparing the practicality of bedside upper airway USG to confirm ETT site after intubation in contrast to capnography in patients of various ages, ethnic groups, and intubation indications. Likewise, a wide range of patients in terms of their age, BMI (less than 30.0 kg/m^2^), and types of operations were included in our study. Adi et al. [19] emphasized the value of USG in excluding esophageal intubation as well. They demonstrated that upper airway USG can accurately determine if a tube is in the trachea or the esophagus while simultaneously observing the upper airway. Their study highlights how crucial it is for emergency physicians to receive proper training in airway USG so they can perform it as a point-of-care procedure on patients who arrive in an emergency. In our study, a single USG-trained anesthesiologist had done the upper airway USG in all the 75 patients included in group U. Application of USG for airway management has been increasing in recent years, hence training USG can help anesthesiologists in elective and emergency settings.

Chun et al. [20] assessed the use of a portable hand-held USG machine to ensure the proper ETT insertion. During all stages of airway management, they bilaterally documented the visceral parietal pleural interface (VPPI) on the chest wall of the patients. They came to the conclusion that thoracic sonography would prove to be a useful tool for validating ETT insertion, particularly in extreme circumstances where other modalities like capnography might not be available and auscultation might not be practical. Bansal et al. [6], in their study, evaluated upper airway ultrasonography, quantitative waveform capnography, and auscultation on adult patients scheduled for elective procedures. They came to the conclusion that upper airway USG can take the place of EtCO_2_ as the preferred method for confirming proper endotracheal intubation.

EtCO_2_ measures the amount of carbon dioxide in the exhaled air. Our study shows that upper airway USG can be used as a direct technique of real-time viewing of upper airway features to determine where an endotracheal tube is inserted. One of the dreaded complications during ETT insertion is esophageal intubation which has a role in perioperative morbidity and was discovered in our study by USG in 15 s. In conditions with the low pulmonary flow, such as cardiac arrest or severe shock, bronchoconstriction, or other circumstances, such as hypothermia, when EtCO_2_ may be inaccurate and the validity of quantitative capnography is questioned, the upper airway USG images are unaffected [3]. USG is painless, portable, reasonably priced, and radiation-free, and its safety has been established. USG is emerging as a useful tool in the management of the upper airways, particularly in emergency situations and outlying clinics where EtCO_2_ is not always easily available. Therefore, in such circumstances as an emergency, upper airway USG can be utilized to verify the insertion of an ETT.

Both methods were safer, quicker, and more efficient than alternative methods like chest radiography. The advantage of upper airway USG is that it is quicker and has a faster confirmation time than EtCO_2_. This study, therefore, shows that upper airway USG could be utilized as the initial verification method of ETT insertion with good reliability.

## Conclusions

Confirmation of ETT placement is vital in any elective/emergency because failure to confirm esophageal intubation increases the chances of peri-operative morbidity. Compared to EtCO_2_, upper airway USG has better reliability, portability, and faster confirmation times of ETT placement. According to our study, Upper airway USG had shown that it has a quicker time of confirmation than EtCO_2_ and can be used for correct identification of esophageal/tracheal intubation. Hence it can be employed as an initial method of verification of ETT placement. Upper airway USG has the potential to replace other non-invasive airway assessment methods as the primary method in the future for the confirmation of ETT placement under general anesthesia.
